# Functional Group Distribution of the Carrier Surface Influences Adhesion of *Methanothermobacter thermautotrophicus*

**DOI:** 10.1155/2020/9432803

**Published:** 2020-01-22

**Authors:** Masaki Umetsu, Takaaki Sunouchi, Yasuhiro Fukuda, Hideyuki Takahashi, Chika Tada

**Affiliations:** ^1^Graduate School of Agricultural Science, Tohoku University, Miyagi 989-6711, Japan; ^2^Research Fellow of the Japan Society for the Promotion of Science (JSPS), Tokyo 102-0083, Japan; ^3^Graduate School of Environmental Studies, Tohoku University, Miyagi 980-8579, Japan

## Abstract

Various support carriers are used for high-density retention of methanogenic archaea in anaerobic wastewater treatment systems. Although the physicochemical properties of carrier materials and microorganisms influence the adhesion of methanogenic archaea, details about the underlying mechanism remain poorly characterized. We applied seven types of chemical surface modifications to carbon felts to clarify the adhesion properties of *Methanothermobacter thermautotrophicus*, a representative thermophilic hydrogenotrophic methanogen. The relationship between carrier surface properties and methanogen adhesion was evaluated. *M. thermautotrophicus* adhesion was significantly increased up to 2.6 times in comparison with control on carbon felts treated with NaOH, HCl, H_2_SO_4_, or Na_2_HPO_4_. Treated carbon felts showed a lower water contact angle, but no correlation between the carrier surface contact angle and methanogen adhesion was observed. On the other hand, at the surface of the carrier that showed improved adhesion of methanogens, the ratio of -COOH : -OH was 1 : 0.65. Such a ratio was not observed with treated carriers for which methanogen adhesion was not improved. Therefore, in the adhesion of *M. thermautotrophicus*, the functional group abundance was important as well as physical surface properties such as the hydrophobicity. Hydrogenotrophic methanogens are involved in active methanation during the startup of anaerobic digestion. Additionally, these methanogenic archaea function as methanogenic cathode catalysts. Therefore, anaerobic digestion performance will greatly improve by controlling the adhesion of hydrogenotrophic methanogens such as *M. thermautotrophicus*.

## 1. Introduction

Anaerobic digestion is considered a sustainable technology compared with aerobic processing, because it incurs minimal costs while producing beneficial end products (such as methane and digestive solution) [1]. As the growth of these methanogenic archaea is slower than that of microorganisms in other consortia, this step represents the rate-limiting reaction in methane production and, in particular, when starting up the reactor [2]. Therefore, having a high density of methanogenic archaea in a reactor is essential to ensure a highly efficient methane fermentation process.

A wide variety of materials (such as glass, pumice, and resins) has been studied as microbial support carriers to achieve high-density retention of the anaerobic consortium. The adhesion of methanogenic archaea is particularly important, and some adhesion experiments using pure culture of the methanogens were performed. Verrier et al. [3] compared the quantity of adhesion of acetotrophic methanogens (*Methanosarcina mazei* and *Methanosaeta concilii*) and hydrogenotrophic methanogens (*Methanobrevibacter arboriphilicus* and *Methanospirillum hungatei*) for hydrophobic different carriers. The condition of the carrier surface that was easy to attach to varied according to the species of methanogens [3]. In addition, although both *Methanosarcina barkeri* and *M. concilii* are mesophilic acetotrophic methanogens, the cell surface properties and favorable carrier surface conditions were reported to be different [4]. However, the parameter influencing the adhesion of methanogens was not identified, because carriers of different materials and surface shape were compared by these experiments. In addition, the adhesion of thermophilic methanogens is not clear, because most of these studies focused on mesophilic acetotrophic methanogens. In anaerobic digestion, acetotrophic methanogens dominated during the stable phase, but hydrogenotrophic methanogen was reported to play an important role during the startup phase [5]. Therefore, by accumulating hydrogenotrophic methanogen on the carrier in the initial phase, it is thought that the startup period of methane fermentation can be shortened and stabilized.

Recently, it was reported that methanogenic archaea generated methane gas by exchanging electrons directly between specific bacteria [6]. The addition of electrically conductive carbon materials [7, 8] and minerals [9, 10] promotes this symbiosis. Accordingly, an electric conductive carrier could support a high-density of methanogenic archaea and significantly improve the performance of anaerobic digestion. Research on such electrochemical relations has advanced in recent years, with studies on methanation using methanogenic archaea as electrode catalysts [11, 12]. In this reaction, methanogens are thought to receive electrons directly from the electrode and produce methane, highlighting the importance of understanding the adhesion of methanogens to electrode materials.

*Methanothermobacter thermautotrophicus* is a typical thermophilic hydrogenotrophic methanogen. *M. thermautotrophicus* strain *Δ*H and *Pelotomaculum thermopropionicum* (a propionate oxidizing bacterium) live together and are connected through a conductive nanowire [13, 14]. In addition, *M. thermautotrophicus* is more likely to be a promising cathode catalyst because *M. thermautotrophicus* is a dominant species on the high temperature methanogenic cathode [12, 15].

This study aimed to clarify the adhesion properties of *M. thermautotrophicus* to conductive materials. Carbon felt was used as the conductive material, and its surface was treated using various chemicals, followed by evaluation of potential change in the contact angle and functional group distribution. In addition, we attempted to clarify the surface properties that participated in the adhesion of methanogen by comparing the quantity of *M. thermautotrophicus* strain *Δ*H that attached to each treated carrier.

## 2. Materials and Methods

### 2.1. Chemical Surface Treatment of Carbon Felt Carrier

Carbon felt (CARBORON® felt; Nippon Carbon Co., Ltd., Japan) prepared in a cylinder shape (1 cm in diameter, 0.5 cm in thickness) was used as a support carrier. The following chemicals were applied to modify the felt surface: KOH, NaOH, HCl, HNO_3_, H_2_SO_4_, Na_2_SO_4_, and Na_2_HPO_4_, while distilled water was used as the control. Different treatments were selected to compare acid treatments (HCl, HNO_3_, and H_2_SO_4_) and basic treatments (NaOH and KOH). We predicted that functional groups containing elements with a high affinity for carbon would show greater attachment to the support. Therefore, treatments containing sulfur (H_2_SO_4_ and Na_2_SO_4_) and phosphorus (Na_2_HPO_4_) with high affinity for carbon were performed.

For each treatment, the carbon felt was completely immersed in a 1.0 M solution of each chemical by multiple vacuum degassing. Chemical surface modification was performed by 6 h of reflux at 100°C using a Findenser and hot stirrer. The support material used for the analysis of surface properties was washed using distilled water until the washing solution became neutral and was then dried in a drying furnace (60°C under atmospheric pressure and humidity). The support carrier used for the adhesion experiment was washed with purified water but without drying.

### 2.2. Analysis of the Surface Properties of the Carbon Felt Carrier

The water contact angle was measured using a contact angle meter (face CA-DS; Kyowa Interface Science Co., Ltd., Saitama, Japan). Fourier-transformed infrared (FT-IR) spectroscopy was used for the analysis of functional groups on the carbon felt material that had been altered by chemical surface modification. The chemically modified carbon felt was cut and mixed with KBr (Wako Pure Chemical Industries, Ltd., Osaka, Japan) at a KBr : carbon felt ratio of 400 : 100 mg. The mixture was analyzed by FT-IR on a Frontier MIR/NIR spectrometer (PerkinElmer Japan Co., Ltd., Kanagawa, Japan).

### 2.3. Adhesion Experiment for *M. thermautotrophicus* Strain *Δ*H

*M. thermautotrophicus* strain *Δ*H (NBRC 100330) was obtained from the NITE Biological Resource Center (NBRC, Chiba, Japan). Culture medium was prepared by dissolving 0.14 g/L of KH_2_PO_4_, 0.54 g/L of NH_4_Cl, 0.20 g/L of MgCl_2_·6H_2_O, 0.15 g/L of CaCl_2_·2H_2_O, 2.5 g/L of NaHCO_3_, 0.20 g/L of yeast extract (Difco, Detroit, MI, USA), 0.80 g/L of sodium acetate, 0.1 mg/L of resazurin, and a trace element solution. Next, 5 mL aliquots of culture medium were transferred to 50 mL serum bottles and sterilized by autoclaving (120°C for 20 min) after bubbling with nitrogen gas (99.999%) for 2 min and then capping with butyl rubber stoppers. Filtered vitamin solution, 0.5 g/L cysteine-HCl, and 0.5 g/L Na_2_S·9H_2_O were injected with a syringe through the rubber stopper, and HCl was added to adjust the pH to 7. The trace element and vitamin solutions were referred to NBRC Medium 1067. The bottles were opened once to insert five treated carriers, bubbled with filtered nitrogen gas for 2 min, and sealed immediately with a butyl rubber stopper.

A 5 mL *M. thermautotrophicus* strain *Δ*H culture aliquot was inoculated into the prepared vial using a syringe. Subsequently, a mixed gas was pumped such that the gas layer had 1.5 atm and a H_2_ : CO_2_ ratio of 80 : 20; stationary culturing was performed at 55°C for 21 days.

### 2.4. Scanning Electron Microscopy (SEM)

After 21 days of culturing, the support carriers were taken out from the culture bottles. The samples were rinsed three times with sterile 0.9% (*w*/*v*) NaCl solution to remove any residue. Cells adhered to the support carrier were fixed with 1.25% glutaraldehyde in 67 mM phosphate-buffered saline (pH 6.5) at 4°C for 3 h [16]. After dehydrating stepwise with ethanol, the samples were dried with *t*-butyl alcohol and coated with platinum palladium. The specimen was then viewed by SEM (SU8000; Hitachi, Tokyo, Japan).

### 2.5. DNA Extraction and Quantitative Real-Time Polymerase Chain Reaction (RT-PCR)

RT-PCR targeting 16S rDNA of archaea was performed to compare the quantity of methanogenic archaea adhered to each treated carrier. After culturing, the carbon felt washed with 0.9% NaCl and was crushed to extract DNA using the PowerSoil® DNA Isolation Kit (MO BIO Laboratories, Inc., Carlsbad, CA, USA) according to the manufacturer's instruction.

RT-PCR was performed using the Chromo 4™ and Opticon Monitor™ software (ver. 3.1; Bio-Rad Laboratories, Inc., Hercules CA, USA) and the Mighty Amp® system for Real-Time (SYBRR Plus) (Takara Bio Inc., Shiga, Japan). According to the manufacturer's instructions, the reaction solution (25 *μ*L) was prepared by mixing 2x Mighty Amp for Real-Time buffer (TB Green™ Plus, 12.5 *μ*L), primers 1106F (5′-TTWAGTCAGGCAACGAGC-3′) and 1378R (5′-TGTGCAAGGAGCAGGGAC-3′; 1 *μ*L each) [17], extracted DNA (1 *μ*L), and sterile Milli-Q water. The RT-PCR program comprised an initial denaturation step at 95°C for 10 s, followed by 50 cycles of denaturation at 95°C for 10 s, annealing at 57°C for 10 s, and extension at 72°C for 6 s [18]. The statistical significance of differences between the carbon felts treated with purified water and chemicals was determined using the unpaired Student's *t*-test.

## 3. Results

### 3.1. Effect of Chemical Treatment on Contact Angle

The contact angle to purified water was measured to compare the surface hydrophobicity of the carbon felt material (Table 1). The contact angle of carbon felt treated with purified water (control) exhibited a high hydrophobicity of 136 ± 6.4°. All other chemical-treated carrier materials showed a tendency towards hydrophilicity; in particular, KOH-, HCl-, HNO_3_-, and H_2_SO_4_-treated carriers exhibited significant hydrophilization (126-130°) (*p* < 0.05, *t*-test). However, even the H_2_SO_4_-treated carrier that possessed the smallest contact angle (126 ± 3.1°) was still within the hydrophobic surface category.

### 3.2. Effect of Chemical Treatment on Surface Functional Groups

**Figure 1:**
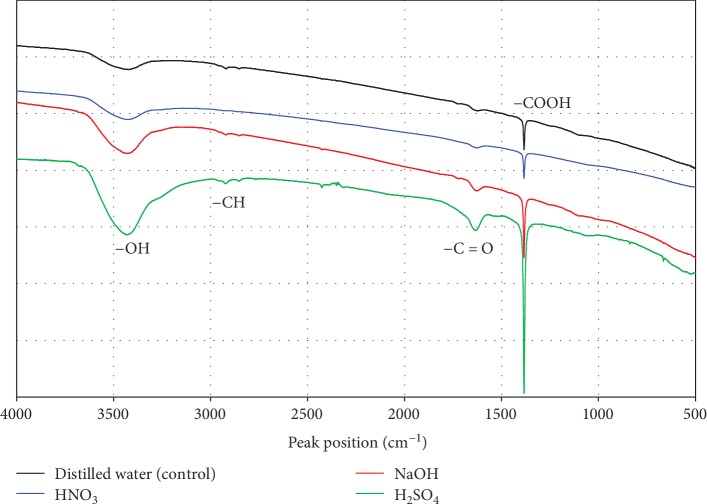
FT-IR spectra (500–4000 cm^−1^) of carbon felts subjected to chemical surface treatment.

FT-IR analysis was performed to investigate the changes in functional groups following surface treatment.  Figure 1 presents the FT-IR spectra (500–4000 cm^−1^) of carbon felts treated with purified water or NaOH, HNO_3_, and H_2_SO_4_. In all samples, bands around 1385, 1630, and 3435 cm^−1^ and bands from 2820 to 2920 cm^−1^ were seen.

Peaks at 1385 cm^−1^ and 1630 cm^−1^ matched those of expansion and contraction of the -COOH and -C=O groups, respectively [19]. The peak at 2820–2920 cm^−1^ matched the peak of -CH. The band at 3435 cm^−1^ matched the peak of expansion and contraction of -OH in the COOH functional group [20, 21]. These findings indicated that the amount of -COOH, -C=O, -CH, and -OH functional groups changed following chemical treatment. This result was common to all eight types of chemical treatments performed in this experiment, and no characteristic change was observed except for peaks caused by these four functional groups.

**Figure 2:**
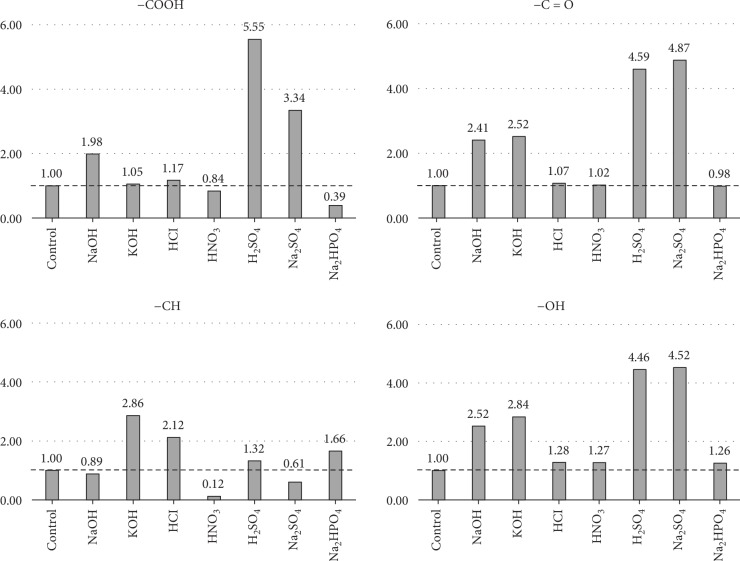
Intensity ratio of different functional groups following chemical surface treatment relative to the control (distilled water).

The intensity ratio of the functional groups on the treated carrier surface relative to the control is shown in Figure 2. In the H_2_SO_4_-treated carbon felt, characterized by the smallest contact angle, the hydrophilic groups -COOH, -C=O, and -OH increased by 5.55 times, 4.59 times, and 4.46 times, respectively, compared with those in the control surface. Both Na_2_SO_4_ and Na_2_HPO_4_ treatments exhibited almost identical contact angles as the control (134°), but the abundance of each functional group differed greatly. This observation demonstrated that the composition of the functional groups could differ in spite of the contact angle remaining the same. No significant difference was noted between the contact angle of NaOH-treated felts and the control; however, the amount of hydrophilic functional groups (-COOH, -C=O, and -OH) was increased by approximately twofold. The quantity of hydrophilic groups showed almost no difference with respect to the control also following acid treatment with HNO_3_, whereas the hydrophobic -CH groups decreased to 1/10.

### 3.3. Changes in Adhesion of Methanogenic Archaea following Chemical Treatment

**Figure 3:**
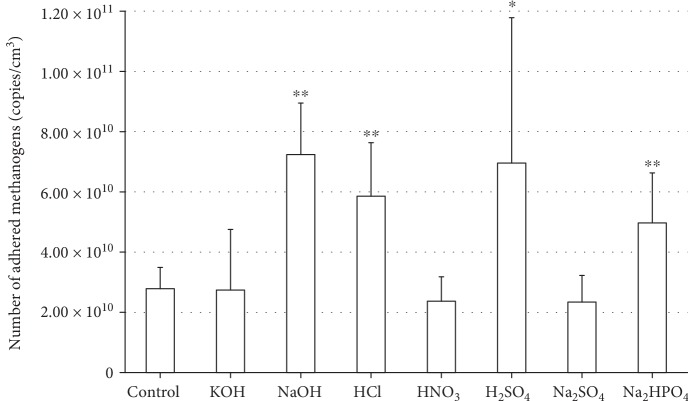
Abundance of *M. thermautotrophicus* strain *Δ*H adhering to the carbon felt after 21 days of culture. Asterisks indicate that the treatment effect is significant (^∗^*p* < 0.05 and ^∗∗^*p* < 0.01) according to Student's *t*-test.

After 21 days of culturing, H_2_ in the gas layer was completely converted to CH_4_ in all culture systems. The quantity of methanogenic archaea adhering to the carbon felts in different systems was compared by quantitative PCR. As shown in Figure 3, attachment was significantly higher in carbon felts treated with NaOH, HCl, H_2_SO_4_, and Na_2_HPO_4_ compared with the control (*p* < 0.05; *t*-test). In contrast, no significant difference was noted between carriers treated with the other chemicals and the control.

**Figure 4:**
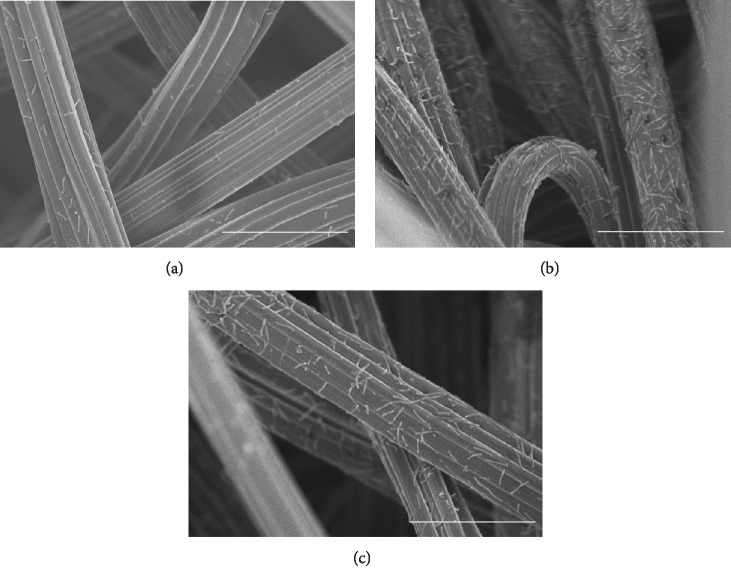
SEM images of carbon felt: (a) distilled water (control), (b) NaOH, and (c) HCl treated. Scale bar = 30 *μ*m.

SEM images in Figure 4 show the methanogens adhering to the chemical-treated carriers. Rod-shaped cells of *M. thermautotrophicus* were found attached to the felt fibers. As a result of having measured quantity of methanogen adhesion per unit area from SEM observation (data not shown), quantity of adhesion significantly increased with NaOH-, HCl-, H_2_SO_4_-, and Na_2_HPO_4_-treated carriers in comparison with the control (*p* < 0.05; *t*-test). SEM evaluation of the changes in adhesion of methanogens in relation to chemical treatment confirmed the results obtained by RT-PCR (Figure 3).

### 3.4. Relationship between the Surface Properties of the Carrier and Adhesion of Methanogenic Archaea

**Figure 5:**
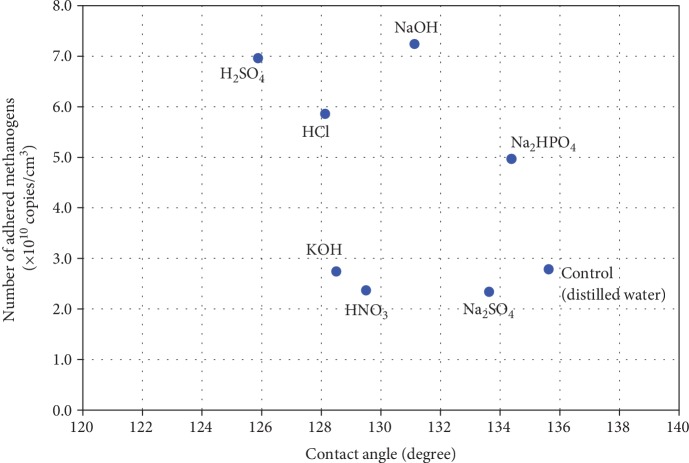
Number of adhered methanogens vs. water contact angle of carbon felts.

As the surface hydrophobicity of the carrier is thought to strongly influence the adhesion of methanogens, the relationship between the contact angle of each treated carrier and the quantity of methanogen adhesion was evaluated (Figure 5). A decrease in contact angle was recorded for all treated carrier surfaces tested in this study relative to the control; however, no correlation was noted between the surface contact angle and adhering quantity of methanogens (*R*^2^ = 0.1688).

**Figure 6:**
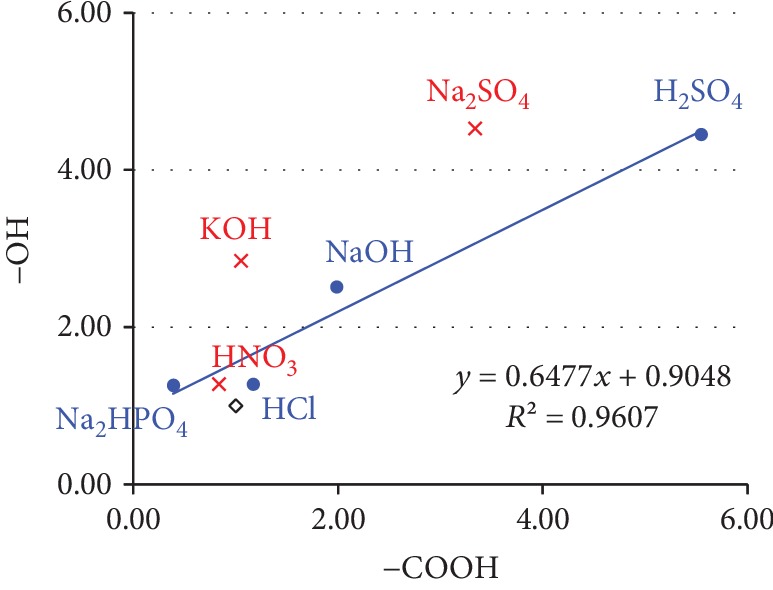
Correlation between -OH and -COOH functional groups on the surface of the carrier (treated with NaOH, HCl, H_2_SO_4_, or Na_2_HPO_4_) wherein quantity of adhesion of methanogen improved. The correlation was determined by linear regression: *y* = *mx*, where *y* = relative amount of -OH and *x* = relative amount of -COOH; *r*^2^ is the coefficient of determination.

Finally, the relationship between the surface functional groups of each carbon felt and adhesion of methanogenic archaea was examined. The amount of the four functional groups (-COOH, -C=O, -CH, and -OH) changed across the seven types of chemical treatment applied in this study. A one-on-one relationship between the abundance of each functional group and adhesion of methanogens was examined; however, no significant correlation was noted for any functional group. Considering that multiple functional groups interact with each other to influence overall surface affinity for methanogens, the relationship between the quantity of methanogens and the -COOH and -OH groups was investigated (Figure 6). With four treated carriers (NaOH, H_2_SO_4_, HCl, and NaH_2_PO_4_), which had a lot of methanogens attached, the -COOH and -OH groups showed stronger correlation (*R*^2^ = 0.9607). In contrast, with the KOH- and Na_2_SO_4_-treated carbon felts, in which the quantity of adhesion did not increase, the correlation of the -COOH and -OH groups was low. These findings suggest that the affinity of the carrier for methanogens was augmented when its surface -COOH and -OH groups were modified in a well-balanced manner. From the degree of the approximate curve and comparison to the standard of functional group distribution of the control, it was thought that it was desirable for a functional group to be distributed in the ratio of ‐COOH : ‐OH = 1 : 0.65.

## 4. Discussion

This study demonstrates that chemical surface treatments of carbon felt material could either increase or decrease the adhesion of the *M. thermautotrophicus* strain *Δ*H. A previous study showed that acid treatment of the carbon brush electrode altered the surface area of the electrode and increased the adhesion of microorganisms [22]. SEM revealed no apparent roughness on the carbon felt surface following any of the chemical treatments conducted in this study (data not shown). Therefore, we believe that no significant changes to the physical appearance of the carrier surface occurred in these experiments.

In general, the cell surface of methanogenic archaea is hydrophobic compared with that of bacteria [23], which makes these cells likely to adhere to more hydrophobic surfaces [24, 25]. Previously, when Verrier compared the quantity of initial adhesion of methanogenic archaea using the polymers with different contact angles, *Methanospirillum hungatei* JFI attached to a hydrophilic surface (water contact angle 58-65°), whereas *Methanosaeta concilii* attached to a hydrophobic polymer (contact angle 90-102°) willingly [3]. Therefore, it is thought that a favorable hydrophobicity condition of the carrier surface varies according to the kind of methanogen species. In this study, the treated carbon felts showed very strong hydrophobicity (water contact angle 126-134°), but *M. thermautotrophicus* attached to all carriers. On the other hand, the KOH-, HCl-, HNO_3_-, and H_2_SO_4_-treated carriers were significantly hydrophilized, but no correlation was observed between the carrier surface contact angle and adhesion of archaea (Figure 5).

Though all carbon felts showed strong hydrophobicity conditions, a difference was seen in the quantity of adhesion of methanogenic archaea after chemical treatment. Therefore, there is a possibility of an interaction between physicochemical surface properties, which potentially influences the adhesion of methanogens. A quantitative comparison of functional groups on the carrier surface using FT-IR revealed that the amounts of -COOH, -C=O, -CH, and -OH groups changed with chemical treatments. In particular, with four treated carriers (NaOH, H_2_SO_4_, HCl, and NaH_2_PO_4_), to which a lot of methanogens were attached, the -COOH and -OH groups existed at a fixed ratio in comparison with the control (*R*^2^ = 0.9607). Generally, carboxyl and phosphate groups are abundant on the surface of microorganisms, where they are believed to promote adhesion through formation of an ester bond with the carrier surface [26]. Thus, it is possible that a well-balanced modification of the -COOH and -OH groups on the carrier surface promoted a chemical bond with *M. thermautotrophicus* strain *Δ*H and improved adhesion. On the other hand, the adhesion quantity of methanogen did not increase although the -COOH and -OH groups were distributed over the HNO_3_-treated carbon felt in the approximate ratio as in the four carriers above. The HNO_3_-treated carrier showed a decreased -CH_3_ group and was 1/10 of that in the control, so the adhesion characteristic of methane bacteria would be clarified in greater detail by evaluating other functional groups and surface properties.

In anaerobic digestion, adhesion experiments with pure methanogenic cultures have focused on acetotrophic methanogens, because these organisms are regarded as playing a dominant role in methane production [4, 27]. Montero et al. reported that hydrogenotrophic methanogens, which were involved in active methanation during the startup phase, were replaced by acetotrophic methanogens when the reactor reached steady-state conditions [5]. Therefore, at the onset of anaerobic digestion, adhesion of hydrogenotrophic methanogens is more important than that of acetotrophic methanogens. In addition, it is thought that hydrogenotrophic methanogens have an important role in direct interspecies electron transfer (DIET) because these methanogens were the dominant species during anaerobic digestion using a conductive carrier and an electrochemical methanogenic cathode. For the effective utilization of methanogenic archaea, it is necessary to evaluate the adhesion of a wide range of methanogenic species as well as acetotrophic methanogens.

The results of the present study suggest that functional groups on the carrier surface contribute to the adhesion of methanogenic archaea. The abundance of functional groups among methanogenic archaea varies greatly because of differences in the cell wall structure among species [28]. Therefore, future studies on the adhesion mechanism of methanogenic archaea should focus on functional group distribution and chemical bonding as well as physical properties on the surface of the carrier and microorganisms. It is thought that adhesion experiments using chemical surface modifications and pure culture are effective at clarifying adhesion properties, such as in the present study. The applicability of anaerobic digestions will greatly improve by controlling the adhesion of methanogenic archaea.

## 5. Conclusions

The amount of *M. thermautotrophicus* strain *Δ*H adhering to the carbon felt surface increased following treatment with NaOH, H_2_SO_4_, HCl, or NaH_2_PO_4_. Chemical treatment changed the hydrophobicity of the carbon felt surface, but no correlation was observed between the carrier surface contact angle and adhesion of archaea. The number of adhering *M. thermautotrophicus* was greater on a carbon felt, whose surface -COOH : -OH ratio was 1 : 0.68. Hydrogenotrophic methanogens are known to have important roles in anaerobic digestion and electrochemistry methanation. Therefore, the performance of anaerobic digestions will improve by controlling the adhesion of hydrogenotrophic methanogen such as *M. thermautotrophicus* using chemical surface modification.

In addition, our results suggested that the functional groups of the carrier surface influenced the adhesion of methanogenic archaea. The adhesion mechanism may become clear in the future by focusing on functional group distribution and chemical bonding as well as physical characteristics.

## Figures and Tables

**Table 1:** Water contact angle of carbon felts after chemical surface treatment.

	Contact angle (degree)^a^
Distilled water	136 ± 6.4
NaOH	131 ± 3.4
KOH	129 ± 3.0^∗^
HCl	128 ± 3.2^∗^
HNO_3_	130 ± 3.0^∗^
H_2_SO_4_	126 ± 3.1^∗^
Na_2_SO_4_	134 ± 3.7
Na_2_HPO_4_	134 ± 3.6

^a^The values are means ± standard deviations (*n* = 16). ^∗^Indicates treatment effect is significant (*p* < 0.05) according to the *t*-test.

## Data Availability

The data used to support the findings of this study are available from the corresponding author upon request.
